# The anatomical evaluation of the dental arches using cone beam computed tomography - an investigation of the availability of bone for placement of mini-screws

**DOI:** 10.1186/1746-160X-9-13

**Published:** 2013-04-20

**Authors:** Feng Pan, Chung H Kau, Hong Zhou, Nada Souccar

**Affiliations:** 1 Department of Orthodontics, Hospital of Stomatology, Xi’an Jiaotong University, Xi’an, Shaanxi, 710004, China; 2 Department of Orthodontics, University of Alabama at Birmingham School of Dentistry, Room 305, 1919 7th Avenue South, Birmingham, 35294, USA

## Abstract

**Objective:**

To assess the amount of maxillary and mandibular inter-radicular bone mass and determine the most reliable mini-screw placement sites.

**Materials and methods:**

Retrospective Cone Beam Computed Tomography (CBCT) images of 40 Angle Class I subjects (20 females, 20 males, aged 16 to 32) were obtained. Measurements on the buccal (BI), medial (MI) and lingual (LI) sides of the inter-radicular spaces were taken at 0, 1, 2, 3, 4, 5 mm from the cemento-enamel junction (CEJ) in an apical direction.

**Results:**

The male and female BI scores ranged from 2.99±0.73 mm to 6.18±1.03 mm and 2.69±0.84 mm to 6.21±1.22 mm respectively. The male and female MI scores ranged from 1.36±0.38 mm to 4.50±0.99 and 1.53±0.66 to 4.77±1.99 mm respectively. LI scores ranged from 2.37±0.70 to 6.47±1.0 mm and 2.45±0.56 mm and 6.66±1.33 mm respectively. In both maxillary and mandibular arch, the inter-radicular space increased in the apical direction except for the buccal and medial inter-radicular spaces between the maxillary first and second molars.

**Conclusion:**

The medial inter-radicular spaces are the decisive parameter for mini-screw placement. In the maxillary arch, regions between central and lateral incisors, lateral incisor and canine, first and second molars are not viable for mini-screw insertion. The residual inter-radicular regions are proper for implantation at 3 mm above the CEJ. In the mandible, the regions between incisors and canines are too narrow for mini-screw insertion and the reliable sites for mini-screws are regions between premolars, molars or first molar and second premolar at 2 mm below the CEJ.

## Introduction

Orthodontic anchorage is defined as “resistance to unwanted tooth movement”. Many intraoral and extraoral appliances have been designed to stabilize a segment of teeth and facilitate tooth movement. Particularly, Temporary Anchorage Devices (TADs) are a collection of attachments that are anchored to bone, and have been reported to provide “absolute anchorage” to achieve optimal tooth movement [1]. They include palatal implants, onplants, mini-plates and mini-screws. A recent systematic review [2] evaluated the survival and failure rates of the different devices over a 12 week period and concluded that palatal implants and mini-plates have superior survival rates than mini-screws. Nevertheless, mini-screws remain very popular among orthodontists, probably because they do not require complicated surgical procedures to be placed and do not depend on patient’s compliance [3]. It is commonly reported that the success rate of mini-screws is as high as 80% [4]. However, many factors can impact the post-surgical stability of mini-screws, such as healing time, the magnitude and direction of applied force, surgical technique, root contact and the site of implantation [5,6]. It has been shown that mini-screws rely on mechanical retention rather than osseointegration, therefore initial stability is an important success factor [7,8]. In order to achieve this initial stability, many studies tried to identify the best sites for mini-screw placement. Schnelle *et al*. [9]have mapped the maxillary and mandibular alveolar bones using panoramic radiographs to determine the most coronal inter-radicular site for mini-screw placement in orthodontic patients, and found the best sites to be mesially to the maxillary first molar and mesially and distally to the mandibular first molar. Since panoramic and periapical radiographs reduce the three-dimensional clinical situation to a two-dimensional image, other researchers tried to investigate the best sites for implant placement using Cone Beam Computed Tomography (CBCT). Kim *et al*. [10] evaluated inter-radicular space in the posterior maxilla and came up with guidelines for mini-implant placement in this area. Monnerat *et al.*[11] found that the best placement site in the mandible was between the first and second molars. In a retrospective study, Park *et al*. [12] evaluated the safety and stability of micro-implant placement by measuring the inter-radicular space, thickness of cortical bone and alveolar process width. Their results indicate that the posterior dentition area in the maxilla and the mandible are safe locations for mini-implant placement.

The objective of the current study is to map the anterior and posterior maxillary and mandibular inter-radicular spaces and to determine the most reliable sites for mini-screw placement using CBCT.

## Materials and methods

### Subjects

Forty CBCT records (20 female, 20 male, aged 16 to 32 years old) were obtained from the Department of Orthodontics patients’ database. Patients were selected according to the following criteria:

•Complete eruption of all second permanent molars

•No missing, rotated, malformed, decayed or restored teeth

•Angle Class I molar relationship

•Healthy periodontal condition

•Crowding less than 5 mm.

•No orthodontic treatment before

The Institutional Review Board of the University of ××××× approved the study and informed consent obtained as part of chart records of the Dental Branch.

### Image device

The Sirona Galileos Cone Beam Imaging Device was used (Sirona Imaging Systems, Charlotte, NC). This system emits radiation doses between 29 μSv to 54 μSv as reported by the manufacturer. It has a scan time of 14 seconds and captures the maxillary-mandibular region in a 180 degree rotation with a radiation-detector configuration. The field of view is a spherical volume of 16 cm and the voxel size is between 0.15 mm to 0.30 mm. The gray scale unit is 12 bit. After scanning, the reconstruction program calculates the entire image volume and the image appears on the screen platform for comprehensive diagnostics. Image manipulation was carried out using the commercially available software, In Vivo Dental 4.1.25.0 (Anatomage, San Jose, CA). Once the image is uploaded, minor orientations to the x, y and z planes were carried out to depict the head position in a natural state or Natural Head Posture (NHP).

### Parameters

**Figure 1:**
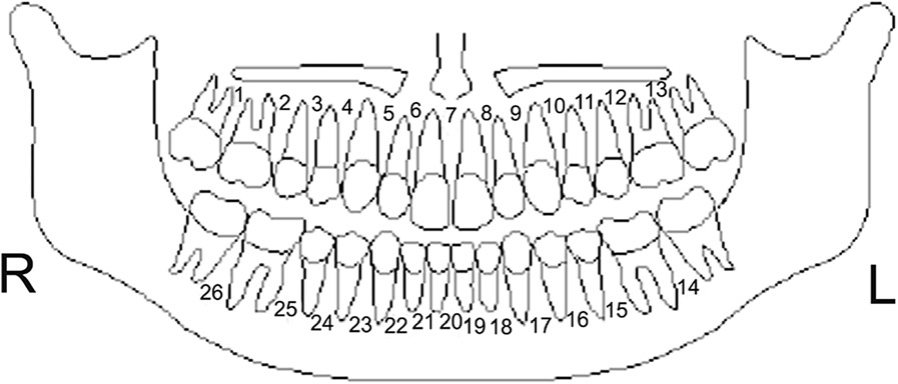
Diagrammatic representation of locations measured.

Twenty-six inter-radicular sites representing potential placement sites for mini-screw were identified (Figure 1). These spaces cover both the anterior and posterior segments of the maxilla and the mandible. Planes were defined in order to cover all three-dimensions of space in the 26 inter-radicular sites:

1. Buccal inter-radicular (BI) space: This measurement is taken from the disto-buccal point of the mesial tooth root to the mesio-buccal point of the distal tooth root.

2. Medial inter-radicular (MI) space: This measurement is taken from the middle point of the distal side of the mesial tooth root to the middle point of the mesial side of the distal tooth root. In case of non-parallel roots, the shortest distance in the mid-area between adjacent roots was chosen as MI.

3. Lingual inter-radicular (LI) space: This measurement is taken from the disto-lingual point of the mesial tooth root to the mesio-lingual point of the distal tooth root.

•The axial plane represents the plane parallel to the cemento-enamel junction (CEJ) between two adjacent teeth.

•The vertical plane is defined as the plane perpendicular to the axial plane in a vertical direction between two adjacent teeth. Using the vertical plane, all measurements were repeated at 6 different locations 1 mm apart in an apical direction.

**Figure 2:**
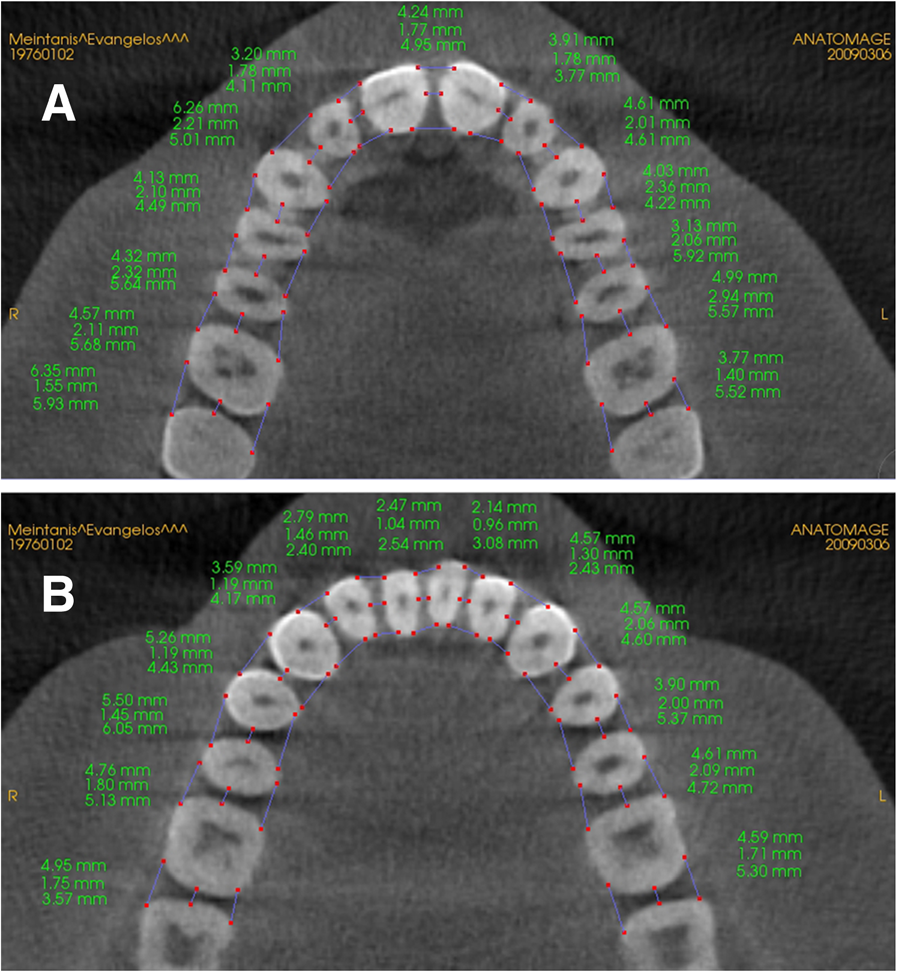
Axial representation of the measurements made for the mandible and maxilla.

•The bucco-lingual plane represents the bucco-lingual direction between two adjacent teeth. Three measurements were taken in the bucco-lingual direction as follows (Figure 2):

Therefore, for every inter-radicular site, three measurements in a bucco-lingual direction were taken and repeated 6 times, one time for each incremental millimeter in the apical direction.

Inter-rater agreement was tested by randomly choosing 10 CBCT images from 10 different patients and repeating the measurements with a two-week interval.

A previous study showed that the distance between mini-screw and dental roots assumed to preserve periodontal health and prevent damage to dental roots is 1 mm around the mini-implant [13]. Meanwhile, other researchers reported that the distance between the screw and the root could not be identified as a risk factor for failure. As long as no contact was present between the root and the mini-screw and the distance to marginal ridge was more than 1.0 mm, the success rate was 100 percent [6]. As the diameter of mini-screws ranges from 1.2 to 2 mm, [6] we considered 2.5 mm of inter-radicular bone mass as an acceptable value and 3.0 mm as a safe value for mini-screw implantation.

### Statistical analysis

The data were analyzed using SPSS 16.0 for Windows (Chicago; SPSS Inc). Independent samples Student’s t-tests was used to check for significant differences between BI vs. LI; BI vs. MI; LI vs. MI and ANOVA was used to check for differences between BI, MI and LI in the six consecutive vertical planes.

## Results

18720 measurements of the tomographic slices were obtained through the 3-D imaging software (InVivoDental version 4.1.35.0). The means and standard deviations of measurement at locations BI, MI and LI at 0-, 1-, 2-, 3-, 4-, 5-mm heights from CEJ are presented in Table 1 (BI), Table 2 (MI) and Table 3 (LI).

**Table 1:** Buccal inter-radicular space (BI) measurements at different heights from CEJ (mean ± SD, mm)

**Site**	**Male**						**Female**					
	**0 mm**	**1 mm**	**2 mm**	**3 mm**	**4 mm**	**5 mm**	**0 mm**	**1 mm**	**2 mm**	**3 mm**	**4 mm**	**5 mm**
**1**	4.91±0.89	4.87±0.90	4.65±0.81	4.30±1.11	3.61±1.09	3.06±1.09	5.54±0.78	5.08±0.91	4.88±1.06	4.21±1.21	3.47±1.13	2.94±1.14
**2**	4.97±0.64	4.80±0.79	4.83±0.69	4.64±0.84	4.71±0.93	4.50±0.95	5.10±0.85	5.00±0.68	4.90±0.66	4.98±0.85	4.96±0.82	4.75±0.96
**3**	5.05±0.55	4.84±0.41	4.80±0.48	4.68±0.50	4.63±0.54	4.69±0.60	5.06±0.61	4.89±0.60	4.90±0.65	4.90±0.64	4.67±0.85	4.50±0.90
**4**	4.81±0.83	4.79±0.83	4.76±0.98	4.81±1.01	4.88±0.90	4.85±0.95	5.04±0.63	5.12±0.74	5.08±0.77	5.10±0.73	4.88±0.76	4.96±0.80
**5**	4.13±0.83	4.34±0.71	4.62±0.55	4.70±0.68	4.93±0.73	5.19±0.71	4.33±0.83	4.57±0.73	4.95±0.90	5.06±0.99	5.01±1.00	5.06±1.07
**6**	4.19±1.08	4.44±1.04	4.16±0.98	4.04±0.91	4.13±0.93	4.25±0.88	4.11±1.05	3.86±0.98	3.85±0.98	4.00±0.86	3.85±0.88	3.77±0.83
**7**	4.52±0.97	5.12±1.00	5.17±1.03	5.15±0.97	5.31±0.84	5.35±0.93	4.77±1.07	4.77±0.85	4.69±0.78	4.93±0.80	4.77±0.82	4.94±0.62
**8**	4.74±1.09	4.78±1.00	4.48±1.01	4.42±0.92	4.50±0.97	4.57±1.18	4.55±1.10	4.50±0.99	4.38±0.84	4.36±0.75	4.31±0.89	4.20±0.78
**9**	4.59±0.88	4.82±0.68	4.95±0.82	4.94±0.72	5.05±0.72	5.28±0.80	4.34±0.80	4.53±1.01	4.58±0.98	4.73±1.07	5.05±1.03	5.24±1.02
**10**	4.89±0.95	5.00±1.01	5.19±0.98	5.23±1.10	5.12±1.16	5.12±1.19	5.17±0.70	5.10±0.75	5.07±0.52	5.47±0.90	5.31±0.99	5.18±1.10
**11**	4.92±0.83	5.05±0.84	4.81±0.91	4.81±0.96	4.71±0.93	4.64±0.80	5.30±0.88	5.18±0.83	5.28±0.79	5.23±0.95	5.11±0.78	5.02±0.78
**12**	4.86±0.75	4.83±0.59	4.81±0.62	4.84±0.71	4.78±0.78	4.76±1.00	5.14±0.78	5.14±0.68	5.11±0.57	5.06±0.61	4.97±1.00	5.14±0.80
**13**	4.72±0.87	4.69±1.00	4.55±1.17	4.31±1.19	3.65±1.27	3.08±1.11	5.36±1.23	5.01±0.94	4.59±0.97	3.99±1.16	3.59±1.28	3.14±1.18
**14**	5.69±0.76	5.65±0.87	5.74±0.90	5.75±1.15	5.80±1.37	5.99±1.23	5.94±1.30	6.25±1.40	6.29±1.54	6.03±1.58	6.04±1.54	6.07±1.84
**15**	5.39±0.82	5.53±1.01	5.65±0.97	5.50±1.14	5.38±0.93	5.22±1.09	5.47±1.12	5.40±1.01	5.65±1.26	5.43±1.37	5.35±1.45	5.61±1.70
**16**	5.59±0.73	5.76±0.93	6.01±0.90	5.93±0.78	6.18±1.03	6.17±1.14	5.62±0.85	5.85±0.81	5.80±0.95	6.02±1.01	6.11±0.85	6.20±0.90
**17**	4.55±0.53	4.68±0.68	4.94±0.56	4.98±0.56	5.11±0.64	4.90±1.10	4.58±0.97	4.61±1.07	4.80±0.90	4.90±1.09	4.93±1.16	4.72±1.20
**18**	4.24±0.88	4.47±0.82	4.54±0.83	4.57±0.82	4.54±0.88	4.64±0.89	3.71±0.79	4.94±0.70	4.22±0.91	4.33±0.88	4.28±0.87	4.21±0.73
**19**	3.53±0.90	3.77±0.83	3.73±0.85	3.83±0.87	3.84±0.91	3.78±1.14	3.01±0.73	3.17±0.63	3.16±0.72	3.11±0.54	2.95±0.77	2.87±0.72
**20**	2.99±0.73	3.13±0.60	3.10±0.68	3.10±0.58	2.99±0.65	3.05±0.62	2.69±0.84	2.85±0.82	2.98±0.76	3.01±0.73	2.95±0.70	2.97±0.66
**21**	3.12±0.82	3.20±0.75	3.32±0.66	3.39±0.65	3.13±0.82	3.27±0.92	2.70±0.69	2.84±0.58	3.04±0.74	2.96±0.65	2.88±0.64	2.98±0.77
**22**	3.37±0.67	3.86±0.74	4.07±0.60	4.04±0.60	4.27±0.64	4.35±0.67	3.15±0.64	3.63±0.64	3.86±0.98	4.02±0.75	3.93±0.80	3.99±0.88
**23**	4.69±0.56	4.59±0.70	4.84±0.72	4.77±0.55	4.76±0.80	4.79±1.07	4.65±0.65	4.75±0.87	5.01±0.99	4.97±1.15	4.94±1.18	4.94±1.17
**24**	5.67±0.61	5.59±0.67	5.80±0.64	5.96±0.75	6.06±0.74	6.14±0.77	5.67±0.75	5.47±0.72	5.64±0.90	5.71±0.85	5.83±0.91	5.63±0.92
**25**	5.50±0.60	5.49±0.97	5.57±0.79	5.42±0.73	5.57±0.75	5.38±0.86	5.84±1.18	5.87±1.50	5.78±1.73	5.93±1.70	5.96±2.10	6.24±2.16
**26**	6.07±0.85	6.07±0.82	5.90±0.86	5.84±1.13	5.82±0.89	5.82±1.17	6.07±0.96	6.21±1.22	6.08±1.33	5.88±1.23	5.79±1.67	5.90±1.66

**Table 2:** Medial inter-radicular space (MI) measurements at different heights from CEJ (mean±SD, mm)

**Site**	**Male**						**Female**					
	**0 mm**	**1 mm**	**2 mm**	**3 mm**	**4 mm**	**5 mm**	**0 mm**	**1 mm**	**2 mm**	**3 mm**	**4 mm**	**5 mm**
**1**	1.75±0.47	2.14±0.54	2.44±0.58	2.52±0.64	2.32±0.73	2.20±0.95	1.95±0.50	2.35±0.40	2.51±0.38	2.62±0.63	2.37±0.78	2.07±0.87
**2**	2.27±0.46	2.54±0.44	2.81±0.47	3.13±0.60	3.21±0.73	3.23±0.88	2.30±0.60	2.67±0.52	3.10±0.54	3.25±0.70	3.40±0.78	3.52±0.90
**3**	2.36±0.34	2.58±0.38	2.85±0.53	3.12±0.51	3.34±0.48	3.37±0.55	2.23±0.41	2.48±0.45	2.81±0.42	3.01±0.42	3.28±0.70	3.36±0.50
**4**	2.31±0.45	2.60±0.48	2.82±0.49	2.91±0.45	3.01±0.52	2.99±0.52	2.43±0.54	2.75±0.64	2.95±0.63	3.14±0.69	3.22±0.77	3.36±0.83
**5**	1.93±0.54	2.17±0.48	2.37±0.49	2.65±0.55	2.76±0.52	2.98±0.59	2.03±0.51	2.34±0.58	2.63±0.60	2.86±0.79	3.15±0.73	3.50±0.87
**6**	1.67±0.57	1.77±0.48	1.88±0.49	1.81±0.50	1.90±0.48	1.94±0.45	1.75±0.49	1.83±0.46	1.89±0.44	1.98±0.51	2.08±0.54	2.08±0.52
**7**	2.20±0.47	2.50±0.57	2.77±0.71	2.97±0.75	3.09±0.71	3.39±0.69	2.35±0.69	2.36±0.62	2.47±0.73	2.70±0.85	2.86±0.80	3.04±0.86
**8**	1.87±0.48	1.93±0.44	1.83±0.48	1.90±0.94	2.11±0.76	2.21±0.73	1.90±0.65	1.96±0.59	2.07±0.60	2.18±0.66	2.35±0.66	2.54±0.64
**9**	2.11±0.57	2.21±0.54	2.40±0.59	2.64±0.57	2.89±0.62	3.16±0.67	2.13±0.70	2.24±0.65	2.38±0.63	2.63±0.64	2.82±0.66	3.06±0.66
**10**	2.24±0.57	2.51±0.46	2.64±0.53	2.79±0.68	2.90±0.61	2.96±0.75	2.50±0.56	2.78±0.57	3.07±0.52	3.23±0.49	3.30±0.52	3.53±0.69
**11**	2.36±0.38	2.67±0.66	2.85±0.43	3.07±0.48	3.34±0.52	3.44±0.60	2.66±0.50	2.89±0.61	3.10±0.44	3.37±0.51	3.56±0.55	3.57±0.6
**12**	2.51±0.56	2.71±0.57	3.02±0.61	3.22±0.64	3.27±0.72	3.27±0.86	2.49±0.65	3.01±0.84	3.22±0.71	3.36±0.78	3.62±0.97	3.52±0.77
**13**	1.77±0.43	2.11±0.52	2.48±0.56	2.65±0.63	2.51±0.73	2.34±0.82	2.00±0.46	2.33±0.45	2.56±0.44	2.56±0.58	2.41±0.76	2.34±0.90
**14**	2.83±0.74	3.40±0.67	3.81±0.80	3.96±0.80	4.10±1.00	4.3±1.10	3.04±0.57	3.59±0.80	4.12±0.93	4.46±1.13	4.48±1.23	4.76±1.28
**15**	2.81±0.46	3.12±0.47	3.46±0.59	3.73±0.60	3.85±0.69	4.05±0.7	2.97±0.64	3.27±0.52	3.63±0.69	3.95±0.86	4.11±1.00	4.34±1.21
**16**	2.61±0.57	2.98±0.54	3.28±0.58	3.69±0.76	3.94±0.84	4.5±0.99	2.72±0.65	3.09±0.78	3.41±0.85	3.72±0.90	4.02±0.88	4.54±1.10
**17**	2.13±0.44	2.40±0.43	2.48±0.45	2.70±0.53	2.81±0.54	3.03±0.68	2.27±0.60	2.48±0.53	2.66±0.63	2.92±0.65	3.05±0.68	3.13±0.72
**18**	1.57±0.56	1.81±0.49	1.94±0.51	2.20±0.52	2.29±0.49	2.58±0.62	1.65±0.64	1.96±0.54	2.07±0.44	2.34±0.51	2.43±0.58	2.70±0.51
**19**	1.48±0.55	1.55±0.45	1.66±0.46	1.75±0.48	1.88±0.50	2.02±0.56	1.53±0.36	1.65±0.43	1.62±0.35	1.63±0.39	1.63±0.38	1.65±0.48
**20**	1.50±0.48	1.66±0.49	1.73±0.50	1.69±0.51	1.72±0.57	1.85±0.63	1.65±0.35	1.64±0.48	1.73±0.48	1.74±0.49	1.83±0.56	1.84±0.56
**21**	1.36±0.38	1.42±0.38	1.53±0.40	1.55±0.41	1.57±0.45	1.70±0.51	1.61±0.41	1.56±0.40	1.54±0.40	1.67±0.34	1.64±0.43	1.61±0.45
**22**	1.37±0.51	1.60±0.56	1.86±0.39	2.08±0.41	2.44±0.52	2.52±0.57	1.64±0.49	1.80±0.49	1.91±0.44	2.10±0.44	2.40±0.55	2.52±0.52
**23**	1.92±0.53	2.19±0.50	2.32±0.46	2.47±0.52	2.64±0.55	2.66±0.65	2.10±0.61	2.32±0.60	2.44±0.59	2.55±0.60	2.78±0.69	2.90±0.77
**24**	2.29±0.42	2.72±0.36	3.20±0.45	3.48±0.52	3.77±0.71	4.06±0.74	2.57±0.69	2.79±0.72	3.10±0.77	3.36±0.77	3.67±0.89	3.83±0.98
**25**	2.59±0.44	2.95±0.55	3.38±0.48	3.58±0.53	3.75±0.49	3.89±0.73	2.85±0.53	3.44±0.71	3.83±1.03	4.15±1.38	4.60±1.70	4.77±1.99
**26**	2.89±0.62	3.52±0.63	3.78±0.74	4.03±0.75	4.12±0.80	4.37±1.03	2.88±0.91	3.54±0.95	3.86±1.10	4.04±1.13	4.18±1.31	4.50±1.41

**Table 3:** Lingual inter-radicular space (LI) measurements at different heights from CEJ (mean±SD, mm)

**Site**	**Male**						**Female**					
	**0 mm**	**1 mm**	**2 mm**	**3 mm**	**4 mm**	**5 mm**	**0 mm**	**1 mm**	**2 mm**	**3 mm**	**4 mm**	**5 mm**
**1**	4.75±0.99	4.90±0.96	5.00±1.06	5.01±1.38	5.00±1.11	5.01±1.13±	5.43±1.36	5.31±1.32	5.25±1.25	5.01±1.26	5.09±1.15	5.03±1.16
**2**	5.25±0.76	5.29±0.80	5.56±0.75	5.83±0.66	5.91±0.75	6.11±0.84	5.28±1.15	5.18±.11	5.54±1.06	5.79±1.14	6.03±1.47	6.35±1.03
**3**	5.67±0.92	5.64±0.92	5.51±0.86	5.43±0.87	5.45±0.88	5.04±1.05	5.34±0.70	5.28±0.68	5.09±0.74	5.01±0.77	4.78±0.76	4.75±0.82
**4**	4.16±0.98	4.02±0.77	3.79±0.78	3.75±0.94	3.59±0.94	3.61±0.80	4.01±1.03	3.96±0.90	3.78±1.04	3.72±0.99	3.78±0.94	3.61±0.81
**5**	4.04±1.02	3.91±1.00	3.98±0.89	3.97±0.80	3.92±0.89	3.96±0.84	4.46±1.42	4.47±1.29	4.49±1.23	4.64±1.18	4.56±1.14	4.64±1.29
**6**	3.29±0.62	3.21±0.74	3.08±0.65	3.06±0.65	3.11±0.80	3.16±0.87	3.85±0.65	3.66±0.70	3.35±0.66	3.27±0.71	3.21±0.72	3.14±0.72
**7**	5.50±1.04	5.44±1.01	5.36±1.00	5.25±0.85	5.20±0.80	5.23±0.74	4.65±1.37	4.42±1.36	4.10±1.21	4.04±1.05	3.98±0.96	4.04±0.95
**8**	3.70±0.75	3.53±0.77	3.33±0.73	3.35±0.67	3.38±0.83	3.47±0.80	3.64±0.88	3.54±0.79	3.52±0.87	3.53±0.84	3.57±0.89	3.60±0.87
**9**	4.53±0.83	4.29±0.10	4.32±0.92	4.29±0.83	4.30±0.87	4.41±0.68	4.17±0.99	4.18±0.97	4.08±0.78	4.31±1.00	4.13±0.77	4.18±0.82
**10**	4.15±0.94	4.01±0.95	3.67±0.87	3.77±0.80	3.70±0.85	3.59±0.86	4.64±1.04	4.27±0.72	4.02±0.84	3.98±0.88	4.00±0.92	3.99±0.82
**11**	5.62±0.49	5.38±0.83	5.43±0.53	5.25±0.78	4.84±1.00	4.88±0.97	5.83±0.73	5.44±0.77	5.34±0.62	5.23±0.57	5.07±0.63	4.91±0.51
**12**	5.29±0.96	5.68±0.85	5.88±0.81	6.16±0.91	6.29±1.04	6.16±1.41	5.45±1.02	5.50±0.91	5.84±0.86	6.26±1.17	6.23±1.36	6.66±1.33
**13**	5.28±1.16	5.13±1.09	5.16±1.00	5.08±1.20	5.13±1.50	5.54±1.35	5.24±1.12	5.19±0.98	5.21±0.77	5.15±0.78	4.84±1.03	4.92±1.04
**14**	5.22±0.73	5.57±0.83	5.63±0.78	5.84±1.02	5.88±0.89	5.80±1.30	5.39±0.85	5.58±0.93	6.00±0.97	6.20±0.91	6.13±1.06	6.24±1.37
**15**	4.93±0.67	5.08±0.68	5.10±0.76	5.23±0.64	4.98±0.84	5.00±0.93	5.13±0.83	5.02±0.89	5.39±0.97	5.40±1.03	5.25±1.07	5.27±1.19
**16**	5.59±0.62	5.83±0.93	5.99±0.87	6.19±0.92	6.20±1.14	6.30±1.23	5.57±0.83	5.72±1.02	5.92±1.06	6.07±1.00	6.16±0.97	5.92±1.39
**17**	5.07±0.84	5.18±0.84	5.28±0.81	5.15±0.93	5.18±0.84	5.09±0.99	4.91±1.08	4.99±1.16	4.92±1.16	4.98±1.09	4.90±1.01	5.08±0.91
**18**	2.97±0.67	2.94±0.69	3.10±0.62	3.03±0.62	2.98±0.55	2.97±0.62	3.29±0.92	3.31±0.90	3.32±0.88	3.28±0.82	3.22±0.86	3.16±0.92
**19**	2.67±0.48	2.65±0.54	2.59±0.58	2.54±0.59	2.51±0.60	2.48±0.67	2.88±0.60	2.78±0.63	2.74±0.60	2.68±0.54	2.42±0.53	2.45±0.56
**20**	3.13±0.87	3.13±0.84	3.08±0.88	3.08±0.88	2.86±0.92	2.79±0.82	2.80±0.65	2.71±0.59	2.78±0.66	2.57±0.72	2.51±0.77	2.46±0.75
**21**	2.84±0.70	2.74±0.68	2.67±0.88	2.56±0.79	2.37±0.73	2.37±0.78	2.98±0.50	2.86±0.58	2.71±0.45	2.51±0.50	2.31±0.62	2.26±0.49
**22**	2.96±0.82	3.02±0.67	3.01±0.70	3.08±0.77	3.07±0.68	2.87±0.67	3.35±0.95	3.18±0.93	3.42±1.05	3.20±0.94	3.32±1.13	3.25±1.34
**23**	4.69±0.71	4.67±0.91	4.67±0.72	4.65±0.92	4.65±0.92	4.53±0.73±	4.26±1.27	4.38±1.18	4.30±1.25	4.42±1.28	4.28±1.46	4.17±1.45
**24**	5.79±0.86	5.86±0.99	6.14±0.87	6.05±1.03	6.42±1.02	6.47±1.07	5.53±0.53	5.64±0.85	5.55±0.93	5.96±1.03	5.77±0.80	5.91±0.96
**25**	4.53±0.76	4.65±0.54	4.89±0.62	5.08±0.65	4.84±0.72	5.08±0.79	5.02±1.02	5.22±0.99	5.40±1.15	5.92±1.18	5.81±1.62	6.07±1.97
**26**	5.01±0.78	5.32±0.82	5.64±0.59	5.63±1.02	5.92±0.99	5.87±0.90	5.06±0.88	5.31±0.77	5.66±1.00	5.74±1.14	5.88±1.40	6.17±1.34

The statistical data analysis showed the following results:

**Figure 3:**
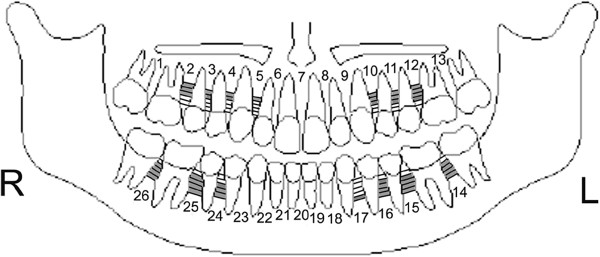
Diagrammatic representation of placement sites for females.

**Figure 4:**
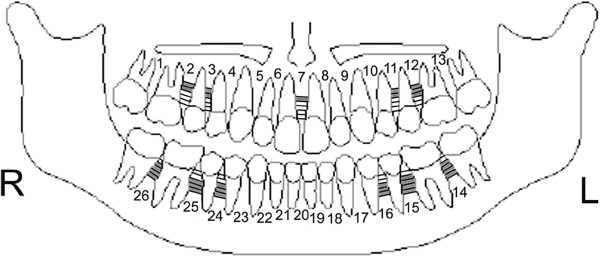
Diagrammatic representation of placement sites for males.

1. BI scores for males and females ranged from 2.99±0.73 mm to 6.18±1.03 mm and 2.69±0.84 mm to 6.21±1.22 mm respectively.

2. MI scores for males and females ranged from 1.36±0.38 mm to 4.50±0.99 and 1.53±0.66 to 4.77±1.99 mm respectively.

3. LI scores for males and females ranged from 2.37±0.70 to 6.47±1.0 mm and 2.45±0.56 mm and 6.66±1.33 mm respectively.

4. Except for some heights in the molar region and in the upper left region between the canines and first premolars in MI, there was no significant difference between males and females in BI, MI and LI of each site (P>0.05).

5. BI and MI were significantly different in both male and female groups (P<0.05).

6. BI an LI were significantly different in both male and female groups (P<0.05).

7. LI an MI were significantly different in both male and female groups (P<0.05).

8. In all the 26 sites of 5 levels (1-, 2-, 3-, 4-, 5-mm from CEJ) measured, the sites for mini-screw placement were classified as No-Go Zones (NGZ), moderate zones (MZ) and excellent zones (EZ). These measurements were 2.5 mm or less, 2.5 to 3 mm and 3 mm or above respectively. The percentage breakdown was tabulated and color coded (Table 4). In general, when considering implant placement, 36.54% were NGZs, 24.23% were MZs and 39.23% were EZs. Schematic charts presenting best sites for implant placement in females and males are presented in Figures 3 and 4 respectively.

9. The measurements at 0-, 1-, 2-, 3-, 4-, 5-mm heights from the CEJ in BI, MI and LI of each site were significantly different (P<0.05).

10. For BI: The maxillary inter-radicular spaces between the first and second molars, the first molar and second premolar, the first and second premolars have a downstream trend in the vertical direction from the CEJ to the apex. The inter-radicular spaces between the lower first molar and second premolar, the lower first and second premolars, the lower first premolar and canine, the upper central incisors had an upstream trend from CEJ to apex.

11. For LI: The inter-radicular spaces between the maxillary first molar and second premolar, the mandibular first and second molars, the mandibular first and second premolars have an upstream trend from the CEJ to the apex. The inter-radicular spaces between the maxillary first and second premolars, the maxilary first premolar and canine, the maxillary central incisors, the mandibular central incisors, the mandibular central and lateral incisors had a downstream trend from CEJ to apex.

12. For MI: With the exception of the region between the maxillary first and second molars (due to buccal root furcation), all sites have a increasing trend from the CEJ to the apex in vertical direction (P<0.05).

**Table 4:** A color index for mini-screw placement sites in alveolar bone

**Kau-pan anatomical map for mini-screw placement**										
**Site**	**Male**					**Female**				
	**1**	**2**	**3**	**4**	**5**	**1**	**2**	**3**	**4**	**5**
**1**	2.14±0.54	2.44±0.58	2.52±0.64	2.32±0.73	2.20±0.95	2.35±0.40	2.51±0.38	2.62±0.63	2.37±0.78	2.07±0.87
**2**	2.54±0.44	2.81±0.47	3.13±0.60	3.21±0.73	3.23±0.88	2.67±0.52	3.10±0.54	3.25±0.70	3.40±0.78	3.52±0.90
**3**	2.58±0.38	2.85±0.53	3.12±0.51	3.34±0.48	3.37±0.55	2.48±0.45	2.81±0.42	3.01±0.42	3.28±0.70	3.36±0.50
**4**	2.60±0.48	2.82±0.49	2.91±0.45	3.01±0.52	2.99±0.52	2.75±0.64	2.95±0.63	3.14±0.69	3.22±0.77	3.36±0.83
**5**	2.17±0.48	2.37±0.49	2.65±0.55	2.76±0.52	2.98±0.59	2.34±0.58	2.63±0.60	2.86±0.79	3.15±0.73	3.50±0.87
**6**	1.77±0.48	1.88±0.49	1.81±0.50	1.90±0.48	1.94±0.45	1.83±0.46	1.89±0.44	1.98±0.51	2.08±0.54	2.08±0.52
**7**	2.50±0.57	2.77±0.71	2.97±0.75	3.09±0.71	3.39±0.69	2.36±0.62	2.47±0.73	2.70±0.85	2.86±0.80	3.04±0.86
**8**	1.93±0.44	1.83±0.48	1.90±0.94	2.11±0.76	2.21±0.73	1.96±0.59	2.07±0.60	2.18±0.66	2.35±0.66	2.54±0.64
**9**	2.21±0.54	2.40±0.59	2.64±0.57	2.89±0.62	3.16±0.67	2.24±0.65	2.38±0.63	2.63±0.64	2.82±0.66	3.06±0.66
**10**	2.51±0.46	2.64±0.53	2.79±0.68	2.90±0.61	2.96±0.75	2.78±0.57	3.07±0.52	3.23±0.49	3.30±0.52	3.53±0.69
**11**	2.67±0.66	2.85±0.43	3.07±0.48	3.34±0.52	3.44±0.60	2.89±0.61	3.10±0.44	3.37±0.51	3.56±0.55	3.57±0.6
**12**	2.71±0.57	3.02±0.61	3.22±0.64	3.27±0.72	3.27±0.86	3.01±0.84	3.22±0.71	3.36±0.78	3.62±0.97	3.52±0.77
**13**	2.11±0.52	2.48±0.56	2.65±0.63	2.51±0.73	2.34±0.82	2.33±0.45	2.56±0.44	2.56±0.58	2.41±0.76	2.34±0.90
**14**	3.40±0.67	3.81±0.80	3.96±0.80	4.10±1.00	4.3±1.10	3.59±0.80	4.12±0.93	4.46±1.13	4.48±1.23	4.76±1.28
**15**	3.12±0.47	3.46±0.59	3.73±0.60	3.85±0.69	4.05±0.7	3.27±0.52	3.63±0.69	3.95±0.86	4.11±1.00	4.34±1.21
**16**	2.98±0.54	3.28±0.58	3.69±0.76	3.94±0.84	4.5±0.99	3.09±0.78	3.41±0.85	3.72±0.90	4.02±0.88	4.54±1.10
**17**	2.40±0.43	2.48±0.45	2.70±0.53	2.81±0.54	3.03±0.68	2.48±0.53	2.66±0.63	2.92±0.65	3.05±0.68	3.13±0.72
**18**	1.81±0.49	1.94±0.51	2.20±0.52	2.29±0.49	2.58±0.62	1.96±0.54	2.07±0.44	2.34±0.51	2.43±0.58	2.70±0.51
**19**	1.55±0.45	1.66±0.46	1.75±0.48	1.88±0.50	2.02±0.56	1.65±0.43	1.62±0.35	1.63±0.39	1.63±0.38	1.65±0.48
**20**	1.66±0.49	1.73±0.50	1.69±0.51	1.72±0.57	1.85±0.63	1.64±0.48	1.73±0.48	1.74±0.49	1.83±0.56	1.84±0.56
**21**	1.42±0.38	1.53±0.40	1.55±0.41	1.57±0.45	1.70±0.51	1.56±0.40	1.54±0.40	1.67±0.34	1.64±0.43	1.61±0.45
**22**	1.60±0.56	1.86±0.39	2.08±0.41	2.44±0.52	2.52±0.57	1.80±0.49	1.91±0.44	2.10±0.44	2.40±0.55	2.52±0.52
**23**	2.19±0.50	2.32±0.46	2.47±0.52	2.64±0.55	2.66±0.65	2.32±0.60	2.44±0.59	2.55±0.60	2.78±0.69	2.90±0.77
**24**	2.72±0.36	3.20±0.45	3.48±0.52	3.77±0.71	4.06±0.74	2.79±0.72	3.10±0.77	3.36±0.77	3.67±0.89	3.83±0.98
**25**	2.95±0.55	3.38±0.48	3.58±0.53	3.75±0.49	3.89±0.73	3.44±0.71	3.83±1.03	4.15±1.38	4.60±1.70	4.77±1.99
**26**	3.52±0.63	3.78±0.74	4.03±0.75	4.12±0.80	4.37±1.03	3.54±0.95	3.86±1.10	4.04±1.13	4.18±1.31	4.50±1.41

No go zones.

Moderate Zones.

Excellent Zones.

## Discussion

This study aims at mapping the maxillary and mandibular inter-raducular spaces using CBCT in order to identify the most reliable sites for mini-screw placement. Our approach differs from other published studies in that the anterior and posterior areas of the maxillary and mandibular arches were examined. Measurements were taken between the roots of two adjacent teeth in the buccal, medial and lingual areas at 6 different heights in the apical direction.

According to Melsen *et al.*, [14] mini-screws placed in non-keratinized tissue have the highest rate of mobility. This finding is corroborated by Turley *et al.*[15] who also suggested placing mini-implants in keratinized tissues. The mean width of attached gingiva in the maxilla and the mandible has been documented in males and females [16]. It varies from tooth to tooth and individual to individual, but is usually less than 5 mm. At same time, the height of alveolar bone process is another decisive factor for mini-screw insertion. Kallestal *et al*. [17] measured the normal distance between CEJ and the alveolar bone crest (ABC) of 30 eighteen years old patients, and found the mean distance of CEJ-ABC in maxilla amounted to 0.9 or 1.0 mm, as well as 0.7 or 0.8 mm in the mandible. Levin [18] compared the CEJ-ABC distance of first molar between adult smokers and nonsmokers and the results showed in nonsmoker group, for the upper first molar, the mean distances of CEJ-ABC were 0.73 mm (right) and 0.80 mm (left); whereas for the lower arch, the mean distances were 1.14 mm (left) and 1.07 mm (right). Bergstrom *et al*. [19] evaluated the premolars and molars periodontal bone height using CEJ-PBC (periodontal bone crest) distance in snuff users and never-users. The results showed that in never-users group, the mean CEJ-PBC distance was 1.06 (0.9-1.16) mm. In the present study, due to the selected subjects having a healthy periodontal condition, we estimate 1 mm as the normal CEJ-ABC distance. According to these estimates, and in order to have the mini-screw placed in attached gingiva, we set the CEJ as a reference level, the measurements of inter-radicular space in this study were taken in incremental millimeters at 0, 1, 2, 3, 4 and 5 mm from the CEJ in an apical direction, and the alveolar bone mass was evaluated at 1, 2, 3, 4 and 5 mm levels for identifying the most reliable sites for mini-screw placement.

The inter-radicular distance is an important parameter to take in consideration when placing a mini-screw. Previous studies have evaluated the amount of bone mass, and made recommendation as to the best placement sites [10,11,13]. Park *et al*. [12] evaluated the safe locations for micro-implant placement from the buccal and the palatal sides of the posterior maxillary and mandibular alveolar bones. They recommend mini-screw insertion between the second premolar and the first molar in the maxillary buccal alveolar bone, between the molars in the maxillary palatal alveolar bone, and inter-radicular spaces from the first premolar to the second molar in the mandibular buccal alveolar bone. The results of the current study show that the ideal sites for mini-implants in the upper and lower arch are slightly different. In the maxillary arch, the regions between first and second molars, lateral incisor and canine, central and lateral incisors are too narrow insert mini-screws. However, it is safe to place mini-screws in the residual inter-radicular regions at 3 mm height or above. In the mandible, the regions between incisors and canines are too narrow to do implantation and other regions are proper for mini-screws at 2 mm height or above. Moreover, with the exception of the buccal and medial distances between the roots of the maxillary first and second molars, inter-radicular spaces have an increasing stream in vertical direction The discrepancy between the current results and the previous reports’ might be due to methodology differences.

Our results showed that in both male and female groups, the buccal and lingual distances (BI, LI) between adjacent roots have statistically significant differences compared with the medial ones (MI) (P<0.05). More often, panoramic or peri-apical projections give a false sense of sending to the amount space available because the images are taken on a projected pathway and are dependent on the focal trough. Consequently, it is very important to evaluate the MI distance on a CBCT because it differs from the BI and LI distances that can be evaluated clinically. Furthermore, since the tip of the mini-screw eventually reaches the MI area, this area has to be wide enough to avoid any periodontal ligament or root damage. In addition, the MI values were greater in females than in males, suggesting larger inter-radicular space leading to a safer mini-screw insertion in females.

This study used CBCT technology to make 3-dimensional measurements in the inter-radicular mesiodistal and vertical directions. Further studies are needed to focus on the bucco-lingual direction and other aspects connected to mini-screw stability.

## Conclusions

•CBCT is a reliable method to evaluate inter-radicular bone mass for mini-screw placement.

•The medial inter-radicular space is the decisive parameter for mini-screw insertion.

•In the maxillary arch, the sites between the central and lateral incisors, lateral incisor and canine, first and second molars are not viable for mini-screw insertion. The residual inter-radicular regions are proper for implantation at 3 mm above the CEJ.

•In the mandibular arch, the sites between incisors and canines are too narrow for mini-screw insertion. The reliable sites for mini-screw placement are between premolars, molars or first molar and second premolar at 2 mm below the CEJ.

•Inter-radicular spaces increase in the vertical direction except for the buccal and medial distances between the roots of the upper first and second molars.

## Competing interests

The authors declare that they have no competing interests.

## Authors’ contributions

FP collected the data and did the analysis. CHK analyzed the data, created the idea, provided funding and helped with manuscript. HZ provided funding for the post-doc and helped with the manuscript. NS helped in the editing of the manuscript. All authors read and approved the final manuscript.
